# Anti-domain 1 β2 glycoprotein antibodies increase expression of tissue factor on monocytes and activate NK Cells and CD8+ cells in vitro

**DOI:** 10.1186/s13317-020-00128-y

**Published:** 2020-03-02

**Authors:** Gayane Manukyan, Anush Martirosyan, Ludek Slavik, Sona Margaryan, Jana Ulehlova, Zuzana Mikulkova, Antonin Hlusi, Tomas Papajik, Eva Kriegova

**Affiliations:** 1 Laboratory of Molecular and Cellular Immunology, Institute of Molecular Biology NAS RA, 7 Hasratyan St., 0014 Yerevan, Armenia; 2 Department of Immunology, Faculty of Medicine and Dentistry, Palacky University Olomouc and Faculty Hospital, Olomouc, Czech Republic; 3 Department of Hemato-oncology, Faculty of Medicine and Dentistry, Palacky University Olomouc and Faculty Hospital, Olomouc, Czech Republic

**Keywords:** Antiphospholipid antibodies, Anti-domain 1 β2GPI, Plasma pool, Primary immune cells, Monocytes, NK cells, Neutrophils, B cells, T cells

## Abstract

**Background:**

β2-Glycoprotein I (β2GPI) represents the major antigenic target for antiphospholipid antibodies (aPL), with domain 1 (D1) being identified as a risk factor for thrombosis and pregnancy complications in APS. We aimed to analyse the ability of aPL, and particularly anti-D1 β2GPI, to stimulate prothrombotic and proinflammatory activity of immune cells in vitro.

**Methods:**

Peripheral blood mononuclear cells (PBMCs) from 11 healthy individuals were incubated with: (1) “anti-D1(+)”—pooled plasma derived from patients suspected of having APS contained anticardiolipin antibodies (aCL), lupus anticoagulant (LA), anti-β2GPI and anti-D1 β2GPI; (2) “anti-D1(−)”—pooled plasma from patients suspected of having APS contained aCL, LA, anti-β2GPI, and negative for anti-D1 β2GPI; (3) “seronegative”—negative for aPL.

**Results:**

The presence of anti-D1(+) and anti-D1(−) plasma resulted in increased HLA-DR and CD11b on monocytes. While only anti-D1(+) plasma markedly increased the percentage and median fluorescence intensity (MFI) of CD142 (tissue factor, TF) on monocytes in comparison with those cultured with anti-D1(−) and seronegative plasma. Anti-D1(+) plasma resulted in increased percentage and MFI of activation marker CD69 on NK and T cytotoxic cells. Expression of IgG receptor FcγRIII(CD16) on monocytes and NK cells was down-regulated by the anti-D1(+) plasma.

**Conclusions:**

Taking together, our study shows the ability of patient-derived aPL to induce immune cell activation and TF expression on monocytes. For the first time, we demonstrated the influence of anti-D1 β2GPI on the activation status of monocytes, NK and cytotoxic T cells. Our findings further support a crucial role of D1 epitope in the promotion of thrombosis and obstetrical complications in APS.

## Background

Antiphospholipid antibodies (aPL) is a heterogeneous family of autoantibodies directed primarily toward phospholipid binding proteins or single plasma proteins [1]. Among a wide variety of known aPL, the clinical significance of anticardiolipin (aCL), anti-β2-glycoprotein I (anti-β2GPI) and lupus anticoagulant (LA) is well established [2]. Transient generation of aPL may be associated with various pathological conditions such as systemic lupus erythematosus (SLE), rheumatoid arthritis, Sjögren’s syndrome, infectious diseases, neurological or cardiac complications, and even in 1–5% of general healthy population [1]. The continuous presence of “classical” aPL, specifically anti-β2GPI, aCL and LA, is a hallmark of the antiphospholipid syndrome (APS), that is the most common cause of acquired thrombophilia, associated with venous and/or arterial thrombosis and pregnancy complications [3]. Numerous epidemiological, clinical, and experimental evidences suggested a positive association between aPL and thrombotic complications in APS patients [4]. In particular, LA and anti-β2GPI are commonly recognised as strong risk factors for thromboembolic events [5].

There are multiple mechanisms on how aPL may catalyse clotting reactions including direct interaction with proteins involved in the initiation and control of blood coagulation, namely β2-GPI, prothrombin, protein C and S, annexin V, factor XII, etc. [6]. Activation of endothelial and immune cells by aPL that have a strong binding ability to cellular membranes is another important thrombogenic mechanism [7]. β2GPI/anti-β2GPI complexes on monocytes activate p38 MAPK and ERK1/2 signalling pathways which result in NF-κB translocation and expression of proinflammatory and prothrombotic molecules, particularly tissue factor (TF) [7] There is a growing body of evidence supporting the key role of aPL-mediated expression of TF on monocytes for a hypercoagulable state in APS [6].

While significant progress has been made toward understanding the pathogenic mechanisms that underlie prothrombotic potential of aPL, an enigma is that only certain aPL-positive individuals develop clinical events [8]. Estimated incidence of aPL among SLE patients range from 11 to 86%, and may be as high as 94% in patients with human immunodeficiency virus (HIV) or chronic hepatitis C virus (HCV) infected patients. Despite this, the occurrence of thromboembolic events in these patients is uncommon [9–11]. In recent years, it has been suggested that aPL titers, isotype distribution, single/double/triple positivity, “autoimmune” and “post-infectious” origin may influence over the ultimate outcome [12, 13]. Therefore, a clear understanding of the heterogeneity within the aPL population is required to establish prospective prediction markers that can be applied to stratify patients as having a low or high probability of thrombotic episodes or other clinical complications.

Central pathogenic importance of antibodies against β2GPI, an abundant plasma glycoprotein composed of five domains, is generally recognised. Evidences suggested that target location may widely determine the pathogenic potential of anti-β2GPI antibodies. In particular, antibodies recognising domain IV/V have been more frequently detected in non-thrombotic autoimmune conditions, whereas anti-β2GPI domain 1 (anti-D1) antibodies are preferentially associated with an increased risk of thrombosis and obstetrical complications [14, 15]. It was proposed that the dimeric form of β2GPI, which has an even greater affinity for anionic phospholipids, is generated when β2GPI is recognised by anti-D1 antibodies [16]. Dimerised complex may then compete with annexin A5, disrupting the protective anticoagulant shield and potentially favouring thrombosis [16]. The anti-D1 β2GPI-mediated effects have been proposed on the basis of epidemiological and clinical data, and the direct pathogenicity of this epitope on immune cells has not been studied yet [17, 18]. In the current study, we aimed therefore to analyse the ability of aPL, and particularly anti-D1 β2GPI, to influence the phenotype and activation status of peripheral blood monocytes, NK cells, T and B cells in vitro.

## Methods

### Plasma samples

Plasma samples were received from the Department of Hemato-oncology (Faculty Hospital, Czech Republic) after laboratory screening for aPL positivity (triple positive) in patients suspected of having APS. All plasma samples were collected using sodium citrate as an anticoagulant. Plasma samples were divided into three groups according to the aPL status: (1) “anti-D1(+)”—plasma contained aCL, LA, anti-β2GPI and anti-D1 β2GPI; (2) “anti-D1(−)”—plasma contained aCL, LA, anti-β2GPI and negative for anti-D1 β2GPI; (3) “seronegative”—plasma negative for aPL. Pooled plasma was aliquoted and stored at − 80 °C until use. aCL and β2GPI, including DI anti-β2GPI antibodies, were measured by CLIA kits (Werfen, Barcelona, Spain) as previously reported [19]. The detailed characterisation of the patients is presented in Table 1. The study was approved by the ethics committee of University Hospital and Palacky University Olomouc. Informed consent was obtained from all participants included in this study.

**Table 1:** A detailed demographic and clinical profile of plasma donors

	Group 1 Anti-D1(+)	Group 2 Anti-D1(−)	Group 3 Seronegative
Number of subjects	6	6	6
Female/Male	6/0	5/1	6/0
Age (years)	35.8 (16–82)	57.8 (27–76)	41.1 (30–50)
Clinical records	1/6-SLE 4/5-thrombotic events 1/6-thrombocytopenia	1/6-SLE 5/6-thrombotic events	–
LA (number of positive patients)	5/6	3/6	0/6
aCL (IgG) (U/ml)	267.6 ± 399.1*	61.10 ± 30.51	< 20
aCL (IgM) (U/ml)	67.17 ± 25.84	277.4 ± 263.2	< 20
Anti-β2GPI (IgG) (U/ml)	257.0 ± 220.8	167.9 ± 118.3	< 20
Anti-β2GPI (IgM) (U/ml)	73.83 ± 48.06	243.9 ± 173.7	< 20
Anti-D1 β2GPI (CU/ml)	106.8 ± 159.7	< 20	< 20

* aPL titers presented as mean ± SD

### Culturing of peripheral blood mononuclear cells (PBMC) in the presence of aPL positive or negative plasma

As a source of primary cells, human PBMCs from healthy donors were isolated. 5 ml of venous blood samples from 11 healthy female volunteers (median age 35 years; range 26–50 years) with no history of APS or thrombotic events in their family history were collected into EDTA-coated tubes after completing a questionnaire regarding their health status. PBMCs were isolated from whole blood by the standard Histopaque-1077 (Sigma-Aldrich) density gradient centrifugation method. Cell viability, as assessed by a trypan blue exclusion test and flow cytometry, was > 98% for each sample. Freshly isolated cells were cultured at 2 × 10^6^/ml in RPMI-1640 medium (Sigma-Aldrich) supplemented with 2 mM l-Glutamine, 100 U/ml penicillin and 100 µg/ml streptomycin in sterile polypropylene round-bottom tubes (to reduce monocyte adherence) in the presence of 25% pooled plasma for all three groups separately for 24 h at 37 °C in 5% CO_2_.

### Flow cytometry analysis

Following a cultivation period, PBMCs were harvested by vigorous pipetting with ice-cold PBS, centrifuged at 400×*g* for 10 min, and washed twice with PBS. Afterwards, the cells were aliquoted and were labelled with the following specific fluorochrome-conjugated monoclonal antibodies: anti-CD27-FITC (clone M-T271), anti-HLA-DR-FITC (L243), anti-CD16/56-PE antibody cocktail (UCHT1/3G8 + MEM-188), anti-CD16-PE (3G8), anti-CD24-PE (ML5), anti-CD4-PerCP-Cy5.5 (SK3), anti-CD11b-PerCP-Cy5.5 (ICRF44), anti-CD80-PerCP-Cy5.5 (2D10), CD8-PE/Cy7 (SK1), anti-CD38-PE/Cy7 (HB-7), anti-HLA-G-PE/Cy7 (87G), anti-CD49d-APC (9F10), anti-CD69-APC (FN50), anti-CD142-APC (NY2), anti-CD19-APC-Cy7 (SJ25C1), anti-CD14-APC-Cy7 (HCD14), all BioLegend. Isotype matched FITC, PE, PerCP-Cy5.5, Pe-Cy7, APC and APC-Cy-7-conjugated irrelevant antibodies (BioLegend) were used as negative controls.

Antigen expression was analysed on Novocyte, ACEA Biosciences flow cytometer. Data acquisition was performed using ACEA Novo Express software. Flow cytometry data were analysed using the FlowJo vX0.7 software (Tree Star, Inc, San Carlos, CA). For each experiment, a minimum of 20,000 events of a gated cell population was counted. The main cell populations were identified using a sequential gating strategy after the exclusion of doublets. T helper lymphocytes (CD3^+^/CD4^+^), T cytotoxic lymphocytes (CD3^+^/CD8^+^), NK cells (CD3^−^/CD16^+^/CD56^+^), B lymphocytes (CD19^+^), monocytes (CD14^+^). 7AAD and PI exclusion stains were used for evaluating cell viability. Results are expressed as the percentage and median fluorescence intensity (MFI) of the cells for each examined marker.

### Statistical analysis

Data analysis was performed with GraphPad Prism 5.01 (GraphPad Software, USA). All values are given as means ± standard errors of the means. Normal distribution was checked with Shapiro–Wilk’s W test. Data was analysed by the Friedman test and differences between groups were determined by the Dunn post hoc test. The significance was defined at the level of *P *< 0.05.

## Results

To address the main question of the study and to define cellular responses in response to aPL, we developed an in vitro model which allowed analysing the influence of patient-derived aPL on the phenotype and activation status of monocytes, NK cells, T and B cells. For this, we cultivated PBMCs from healthy individuals with pooled plasma from 3 studied groups separately: anti-D1(+), anti-D1(−), and seronegative, and analysed by flow cytometry as previously described [20].

### Anti-D1 β2GPI induces TF expression on monocytes

**Fig. 1:**
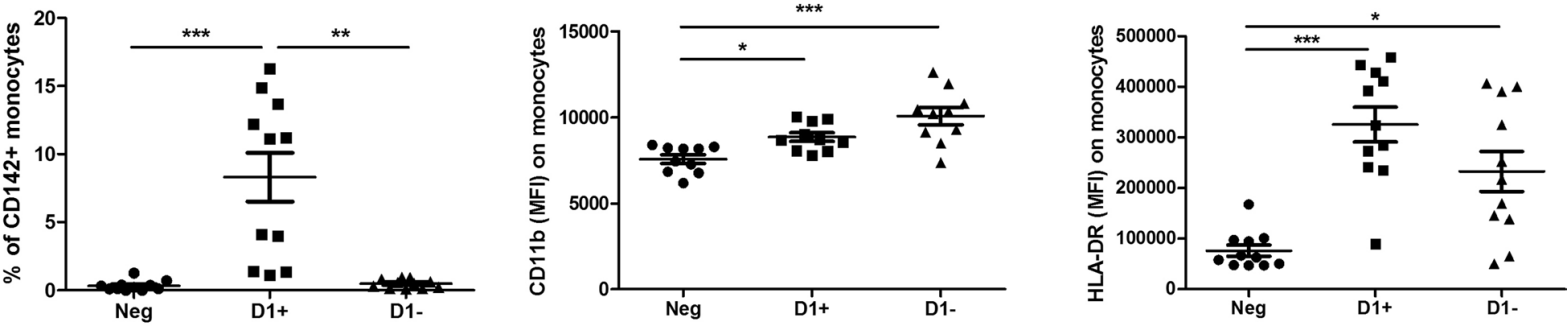
The percentage of CD142 (TF) and MFI of CD11b, and HLA-DR on monocytes after the culturing of PBMCs from healthy subjects with seronegative plasma (Neg), anti-D1(+) plasma (D+), and anti-D1(−) plasma (D−)

Monocytes were initially gated based on size, granularity, and CD14. First, we analysed the expression of thromboplastin CD142 (tissue factor, TF), a multifunctional protein which enables thrombin formation [6]. The percentage and MFI of CD142 were increased on monocytes treated with anti-D1(+) compared to the cells cultured with anti-D1(−) (*P *< 0.01 and *P *< 0.05, respectively) and seronegative (*P *< 0.001 and *P *< 0.001, respectively) plasma (Fig. 1).

### aPL mediate activation of monocytes and NK cells

Next, we showed that the cultivation of PBMCs with both anti-D1(+) and anti-D1(−) plasma resulted in a marked activation of monocytes as defined by the increased expression levels of CD11b (*P *< 0.05 and *P *< 0.001, respectively) and the increased  % of CD11b+ monocytes (*P *< 0.05 and *P *< 0.001, respectively) compared with the monocytes cultured with seronegative plasma. Similarly, the expression of HLA-DR was increased in the anti-D1(+) and anti-D1(−) groups compared to the seronegative one (*P *< 0.001 and *P *< 0.05, respectively) (Fig. 1). The exposure of the cells with aPL did not induce significant differences in the expression levels of HLA-G on monocytes (data not shown).

**Fig. 2:**
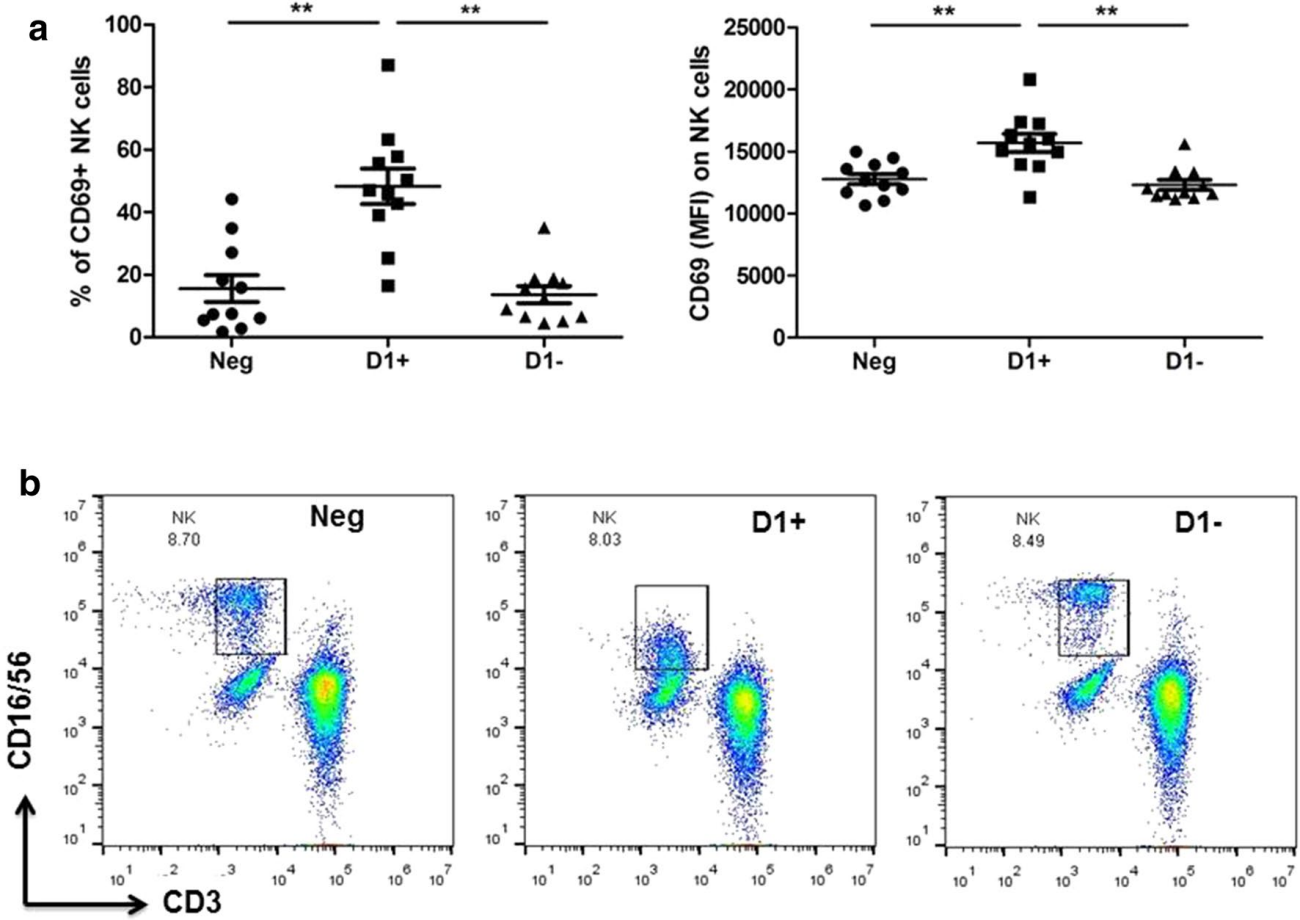
The phenotype of NK cells after the culturing of PBMCs from healthy subjects with seronegative plasma (Neg), anti-D1(+) plasma (D+), and anti-D1(−) plasma (D−). **a** the percentage and MFI of activation marker CD69 on NK cells in all studied groups; **b** representative dot-plots of NK cell (CD3−/CD16+/CD56+) distribution in three studied groups showing CD16/CD56^bright^ phenotype of NK cells in seronegative and anti-D1(−) groups and CD16/CD56^dim^ phenotype of NK cells in anti-D1(+) group

Anti-D1(+) plasma resulted in the prominent activation of NK cells. Namely, the percentage and MFI of activation marker CD69 were increased in the anti-D1(+) group in comparison with anti-D1(−) (*P *< 0.01 and *P *< 0.01, respectively) and seronegative (*P *< 0.01 and *P *< 0.01, respectively) plasma. The percentage of HLA-G + NK cells was increased in anti-D1(+) (*P *< 0.01) and anti-D1(−) (*P *< 0.01) groups comparing seronegative plasma without affecting expression intensity (Fig. 2a).

### Anti-D1 β2GPI substantially reduce phenotype-specific markers CD16 and CD56 on NK cells

As shown in Fig. 2b, a substantial proportion of NK cells treated with anti-D1(+) showed down-regulation of both phenotype-specific markers, CD16 (FcγRIIIA) and CD56 in comparison with anti-D1(−) (*P *< 0.001) and seronegative (*P *< 0.05) groups. It is known that IgG can induce NK and monocyte cell-mediated complement-dependent cytotoxicity (CDC), antibody-dependent cell-mediated cytotoxicity (ADCC) and apoptosis in vivo [21]. To verify whether anti-D1(+) caused a cytotoxic effect mediated by the antibodies, we have analysed, in parallel, the viability of the treated cells. The number of non-viable cells was comparable to those of the cells treated with anti-D1(−) and seronegative plasma (data not shown).

Similar results were observed on monocytes. Particularly, the percentage of monocytes cultured with anti-D1(+) bearing CD16 was tended to be reduced compared to the anti-D1(−) and seronegative groups. In contrast, in the group of anti-D1(−), the percentage of CD16 was increased compared to both anti-D1(+) (*P *< 0.001) and seronegative (*P *< 0.01) groups. Due to the difficulties in defining monocyte subsets after cultivation, we were not able to delineate and analyse monocyte subsets. Despite these difficulties there were apparent differences in the distribution of monocyte subsets (CD14/CD16) in the samples cultured with anti-D1(+) and anti-D1(−) (data not shown). The differences in monocyte phenotypes after culturing with plasma containing aPL deserves further experiments.

### Anti-D1 β2GPI activate T cytotoxic cells and suppress B cells

**Fig. 3:**
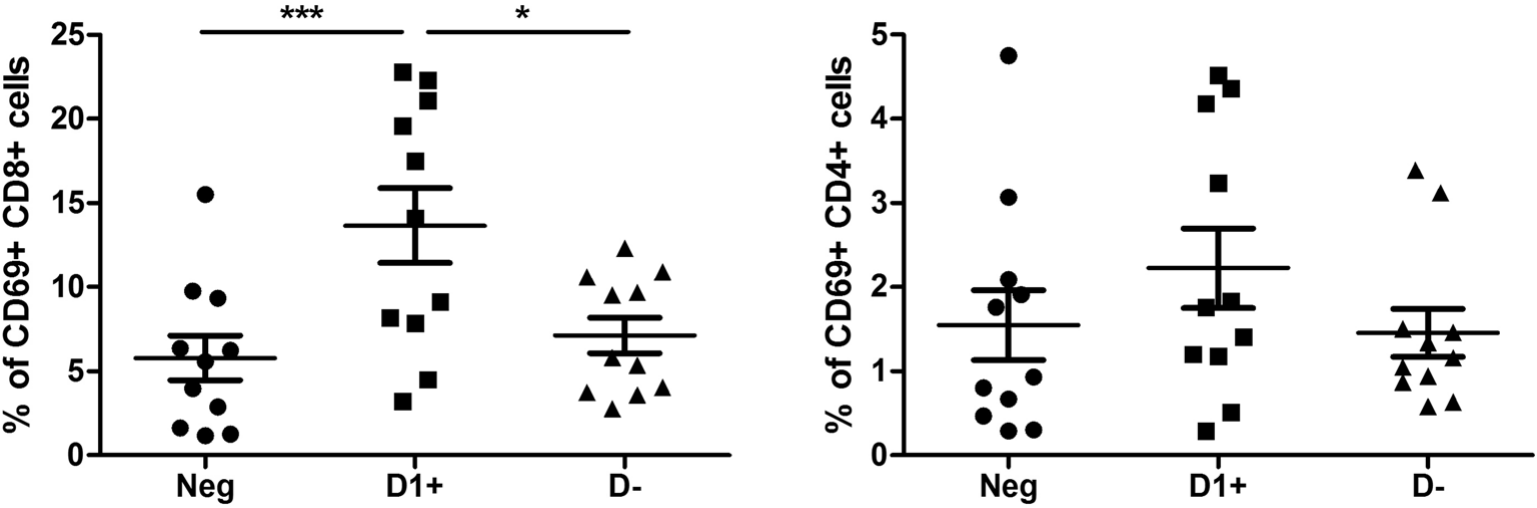
The percentage of activation marker CD69 on CD4+ (**a**) and CD8+ (**b**) T cells after the culturing of PBMCs from healthy subjects with seronegative plasma (Neg), anti-D1(+) plasma (D+), and anti-D1(−) plasma (D−)

**Fig. 4:**
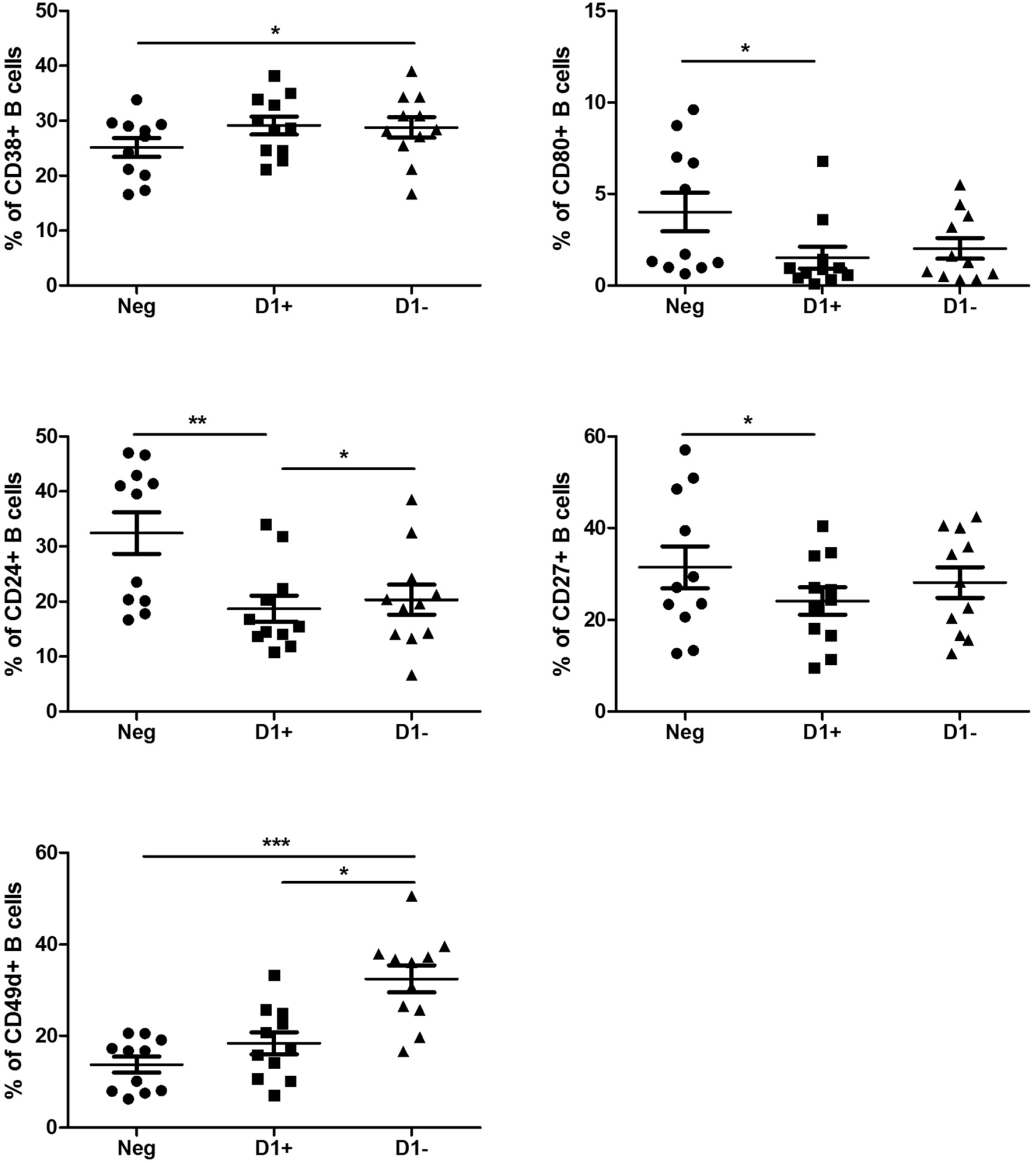
The percentage of CD24, CD27, CD38, CD49d, and CD80 after the culturing of PBMCs from healthy subjects with seronegative plasma (Neg), anti-D1(+) plasma (D+), and anti-D1(−) plasma (D−)

CD4+ (T helper) cells were not affected by any combination of aPL (Fig. 3a). However, CD8+ (T cytotoxic) cells showed a marked increase in the number of CD8+ cells bearing activation marker CD69 in the presence of anti-D1 (+) as compared to the anti-D1(−) (*P *< 0.05) and seronegative (*P *< 0.001) groups. Similarly, CD69 MFI was up-regulated on CD8+ cells in the presence of anti-D1(+) as compared to the anti-D1(−) (*P *< 0.01) and seronegative (*P *< 0.001) groups (Fig. 3b). As a confirmation of the previous studies, we report here that CD4+ and CD8+ T cells express HLA-G at low levels [22]. However, significant changes in the presence of aPL were not observed. In contrast to CD8+ cells, cultivation with anti-D1 (+) resulted in a decreased number of B-cells bearing CD24 (*P *< 0.01), CD27 (*P *< 0.05), and CD80 (*P *< 0.05) as compared to the seronegative one. Cultivation of the cells with anti-D1(−) resulted only in the increased % of CD49d+ (*P *< 0.05) and CD38+ (P < 0.05) B cells as compared to the seronegative group. When the studied B cell markers were compared between anti-D1(+) and anti-D1(−) groups, we observed an increased percentage of CD49d (*P *< 0.05) and CD24 (*P *< 0.05) positive cells in the anti-D1(−) group (Fig. 4).

## Discussion

Although it is believed that anti-D1 β2GPI antibodies play a role in APS, their biological and pathogenic functions are largely unknown. Our study primarily focused on the evaluation of the effects of patient-derived plasma containing aPL, and particularly anti-D1 β2GPI, on activation status of circulating immune cells in vitro. We, for the first time, demonstrated marked activation of monocytes, NK and CD8+ cells in the presence of anti-D1 β2GPI autoantibodies.

In this study, aPL modulated the host immune response, executing pro-inflammatory effects on monocytes as assessed by the increased expression of CD11b and HLA-DR. Despite marked activation of the monocytes caused by the aPL, only anti-D1 β2GPI antibodies induced procoagulant activity of monocytes as assessed by the increased expression of TF on monocytes. Our results confirm the data obtained in epidemiological and clinical studies showing an association of anti-D1 β2GPI with late pregnancy morbidity and thrombosis in APS [23, 24]. The presence of thromboembolic complications is indispensable for the diagnosis of APS [25]. The hypercoagulable state in APS is markedly different from other known hypercoagulable states, and thrombosis can occur in almost every vessel and microcirculation, favouring systemic disbalance which might be driven by autoantibodies [26]. Coagulation and inflammation have a common evolutionary origin and are integrated by a vast crosstalk which interacts in a very complex manner [27]. The central feature of hypercoagulability induced by inflammation is cytokine-mediated TF expression [28]. Our previous data are consistent with the hypothesis that anti-β2GPI antibodies engage TLR4, inducing proinflammatory phenotype in monocytes which could be a risk factor for the onset and progression of the clinical features typical for APS [29, 30]. Whether anti-D1 β2GPI antibodies might represent a potential risk factor for thromboembolic events deserve future investigations.

Although experimental models have shown the main role of thrombotic events in APS, studies in humans suggest that thrombotic events cannot account for all of the histopathologic findings in placenta from women with obstetric APS [3, 26, 31]. aPL may target the trophoblast, through direct binding with anionic PL or β2GPI expressed on the surface of trophoblast cells affecting its invasiveness in vitro, inducing syncytiotrophoblast apoptosis, a complement activating at the maternal–fetal interface, etc. [31–33]. Another mechanism of spontaneous abortion might be NK cell activity [34]. By virtue of their ability to mediate cytotoxicity, NK cells and CD8+ cells are positioned to play a role in regulating autoimmune responses [35]. The two major NK cell subsets, CD56^bright^ and CD56^dim^, exert different functional activities [36]. Within these subsets, antibody-mediated cell cytotoxicity is mostly confined to the CD56^dim^ subset, whereas cytokine production is assigned to the CD56^bright^ subset [37]. Observed in our study CD56^dim^ NK cells in the presence of anti-D1 β2GPI as well as an increase in activation marker CD69 point to the prevalence of NK cells with an increased cytotoxic activity. The shedding of CD16 from the surface of NK cells through antibody cross-linking of activating receptors was shown to produce a potent signal for inducing ADCC [38]. It was shown that proteolytic cleavage or endocytosis of CD16 are aimed to dampen stimulatory signals and control excessive inflammation leading to autoimmunity, representing a regulatory mechanism by which NK cell activity is restricted to avoid auto-aggressiveness [39, 40].

It is believed that cytotoxicity of the NK cells may represent a mechanism of abortion [41]. It was shown that the increased number of NK cells in peripheral blood of APS patients with recurrent spontaneous abortion was correlated with the lower number of CD56^bright^ cells [42, 43]. Increased number of NK cells was also found in placental bed biopsies in recurrent spontaneous abortions, and the cytotoxic activity of these cells has been shown to be increased [44]. Demonstrated changes in the phenotype of NK cells caused by anti-D1 β2GPI in our study might represent a potential mechanism explaining an association of domain D1 with obstetrical complications in APS [23].

A marked increase in cellular responses to anti-D1 β2GPI, namely the activation of monocytes, Tc and NK cells, was associated with the suppressed B cell activation. A shift from a humoral immune response to a cellular immune response in the presence of anti-D1 β2GPI, occurring at least in vitro, may account for deleterious effects during pregnancy. The pathogenicity of autoantibodies in different autoimmune disorders has been extensively debated. The direct pathogenic role of aPL antibodies was demonstrated in animal models showing pregnancy loss and thrombus formation after the immunisation of animals with aPLs [45, 46]. Another proof is evidence of the clinical improvement after the removal of autoantibodies by plasma exchange or plasmapheresis [47, 48]. Our study shows the ability of patient-derived aPL to induce immune cell activation and contribute to the thrombotic events. Functional relevance and signaling pathways involved in the immune response to anti-D1 β2GPI deserve further investigations.

The study has several limitations. First, the main criterion for the patients’ selection was aPL positivity, not diagnosis. Second, the influence of the different treatments was not taken into account. Third, the mean titers of IgG and IgM aPL in studied groups differ. Finally, the pooled plasma preparations certainly contain a different set of humoral factors which could not be predicted and measured, preventing a more precise estimation of the pathogenic role of studied antibodies. Despite this, we believe that cellular responses elicited by the patient-derived plasma may better reflect the situation occurring in vivo than using purified antibodies.

## Conclusions

Our findings further support the concept that anti-D1 β2GPI have the potential to contribute to the thrombotic events and obstetrical complications. As treatment of patients with aPL-associated thrombosis or fetal loss requires the use of several anticoagulation strategies and even immunotherapy, the epitope specificity of circulating aPL may provide a rationale for specific treatment of APS patients according to their aPL profile. Accurate risk stratification for the development of vascular events in patients remains challenging.

## Data Availability

The datasets used and/or analysed during the current study are available from the corresponding author on reasonable request.

## Abbreviations

APS: Antiphospholipid syndrome
aPL: Antiphospholipid antibodies
β2GPI: β2-Glycoprotein I
anti-D1 β2GPI: Anti-domain 1 β2-glycoprotein I antibodies
aCL: Anticardiolipin antibodies
LA: Lupus anticoagulant
TF: Tissue factor
Tc: T cytotoxic cells
NK: Natural killer
ADCC: Antibody-dependent cellular cytotoxicity
TLR: Toll like receptor
SLE: Systemic lupus erythematosus
MAPK: P35 mitogen-activated protein kinases
NF-κB: Nuclear factor kappa-light-chain-enhancer of activated B cells
ERK1/2: Extracellular signal-regulated kinase 1/2
